# DLAD4U: deriving and prioritizing disease lists from PubMed literature

**DOI:** 10.1186/s12859-018-2463-0

**Published:** 2018-12-28

**Authors:** Junhui Shen, Suhas Vasaikar, Bing Zhang

**Affiliations:** 1 Information Center, Beijing University of Chinese Medicine, Beijing, China; 2 Lester and Sue Smith Breast Center, Baylor College of Medicine, Houston, TX USA; 3 Department of Molecular and Human Genetics, Baylor College of Medicine, One Baylor Plaza, Mail Stop BCM600, Houston, TX 77030 USA

**Keywords:** Gene-disease association, Drug-disease association, Literature mining, Web application, Information retrieval

## Abstract

**Background:**

Due to recent technology advancements, disease related knowledge is growing rapidly. It becomes nontrivial to go through all published literature to identify associations between human diseases and genetic, environmental, and life style factors, disease symptoms, and treatment strategies. Here we report DLAD4U (Disease List Automatically Derived For You), an efficient, accurate and easy-to-use disease search engine based on PubMed literature.

**Results:**

DLAD4U uses the eSearch and eFetch APIs from the National Center for Biotechnology Information (NCBI) to find publications related to a query and to identify diseases from the retrieved publications. The hypergeometric test was used to prioritize identified diseases for displaying to users. DLAD4U accepts any valid queries for PubMed, and the output results include a ranked disease list, information associated with each disease, chronologically-ordered supporting publications, a summary of the run, and links for file export. DLAD4U outperformed other disease search engines in our comparative evaluation using selected genes and drugs as query terms and manually curated data as “gold standard”. For 100 genes that are associated with only one disease in the gold standard, the Mean Average Precision (MAP) measure from DLAD4U was 0.77, which clearly outperformed other tools. For 10 genes that are associated with multiple diseases in the gold standard, the mean precision, recall and F-measure scores from DLAD4U were always higher than those from other tools. The superior performance of DLAD4U was further confirmed using 100 drugs as queries, with an MAP of 0.90.

**Conclusions:**

DLAD4U is a new, intuitive disease search engine that takes advantage of existing resources at NCBI to provide computational efficiency and uses statistical analyses to ensure accuracy. DLAD4U is publicly available at http://dlad4u.zhang-lab.org.

**Electronic supplementary material:**

The online version of this article (10.1186/s12859-018-2463-0) contains supplementary material, which is available to authorized users.

## Background

A key aspect of biomedical research is to study genetic, environmental, and life style factors associated with human diseases, symptoms of diseases, and treatment strategies for diseases. Due to recent technology advancements, disease related knowledge is growing rapidly. It becomes nontrivial to find comprehensive answers for simple and common questions such as “Which diseases are associated with the MTHFR gene?” and “Which neoplasms have been treated by pembrolizumab?”. To answer such questions requires not only retrieving relevant publications through the PubMed search engine, but also to read, and prioritize disease lists. The challenge is to prioritize results on-the-fly without losing precision.

To consolidate disease-related knowledge, many databases have been developed. For example, the Online Mendelian Inheritance in Man (OMIM) database provides an authoritative collection of the relationships between human diseases and genes [1]. Gene-disease relationships identified from genome-wide association studies (GWAS) and phenome-wide association studies (PheWAS) have been carefully curated and documented in databases such as the Genetic Association Database (GAD) [2], the GWAS catalog [3], and the PheWAS catalog [4]. The Comparative Toxicogenomics Database (CTD) [5] focuses on the effects of environmental compounds on human health and contains manually curated information about chemical–gene/protein interactions, chemical–disease and gene–disease relationships. These databases are valuable resources for the whole biomedical research community. Nevertheless, due to the explosive growth in biomedical literature, manually curated databases are difficult to update, and incompleteness is becoming a well-recognized problem [6].

Text mining tools have been developed to computationally identify disease-related relationships [7–9]. As an example, COREMINE [10], which is an extension of PubGene, is a public search engine to identify relationships between biomedical terms, including genes, drugs and diseases. COREMINE and similar text mining tools, such as Literature-derived Human Gene-Disease Network (LHGDN) [11] and Bio-Entity Finder and Relation Extraction (BeFree) [12], usually depend on heavy computation, and the accuracy of the resulted disease-query relationships has not been systematically assessed.

In addition to text mining, other methods have also been developed to predict disease-related relationships. CTD infers new relationships using a “guilt-by-association” approach. For example, if gene A shares a curated interaction with chemical C, and chemical C shares a curated association with disease B, an association between gene A and disease B is predicted [13]. Although many new associations could be inferred based on this approach, they may include a large number of false positives.

Here, we describe DLAD4U (Disease List Automatically Derived for You), a web-based tool for disease retrieval and prioritization. DLAD4U is built upon existing resources at the National Center for Biotechnology Information (NCBI) to gain computational efficiency. The simple interface of DLAD4U facilitates intuitive tool usage and easy interpretation of results. We evaluated the quality of the disease lists generated by DLAD4U using manually curated “gold standard” lists and compared the performance of DLAD4U with related tools.

## Methods

### Publication retrieval

DLAD4U uses the eSearch API developed by NCBI to search the MEDLINE database and to retrieve publications on the fly [14]. For each query term, eSearch outputs an XML file with various types of information. The XML file is then parsed to obtain a list of PubMed IDs (PMIDs) associated with the query.

### Disease retrieval

Diseases related to the PMIDs are identified using a precomputed publication-to-disease link table. To build the link table, we first acquired PMIDs for all papers published since January 1, 1960. Next, we used NCBI’s eFetch API [14] to retrieve data records for all PMIDs in the XML format. The XML files include MeSH (Medical Subject Headings), which is the National Library of Medicine (NLM) controlled vocabulary thesaurus used for indexing articles for PubMed. MeSH terms located under C01-C26, F02 and F03 in the MeSH Tree Hierarchy [14] were used to determine publication-to-disease relationships. MeSH terms “Disease”, “Disease Progression”, “Disease Attributes”, and “Disease Models, Animal” were removed because the lack of specificity. The current publication-to-disease link table was built on November 17, 2016 and includes 5013 diseases, 13,000,996 publications, and 13,058,498 publication-to-disease relationships.

### Disease prioritization

DLAD4U uses the hypergeometric test to prioritize retrieved diseases for a query term. For a given query Q and a disease D, let *m* be the total number of publications in the publication-to-disease link table, among which *j* publications involve disease D (i.e., disease-related publications). Let us further assume that *n* out of the *m* publications are retrieved for the query (i.e., query-related publications) and *k* out of the *n* involve disease D. Our method calculates the probability of observing *k* or more disease D-related genes when *n* publications are randomly selected from *m*. Disease *D* is then scored using the following formula:

$$ {S}_D=-{\log}_{10}f\left(m,n,j,k\right), $$

where

$$ \mathrm{f}\left(\mathrm{m},\mathrm{n},\mathrm{j},\mathrm{k}\right)=\sum \limits_{i=k}^{\min \left(n,j\right)}\frac{\left(\begin{array}{c}m-j\\ {}n-i\end{array}\right)\left(\begin{array}{c}j\\ {}i\end{array}\right)}{\left(\begin{array}{c}m\\ {}n\end{array}\right)} $$

For diseases with the same score, we rank them by the number of publications describing the disease in the link table.

### Web implementation

The DLAD4U user interface was developed in HTML and PHP languages based on our previously published GLAD4U framework [15].

### Performance evaluation and comparison

To assess the performance of the DLAD4U algorithm, we used gene and drug/chemical terms as queries. Manually curated gene-disease associations in GAD and gene/drug-disease associations in CTD were used to establish the gold standard.

We downloaded the GADCDC_data.tsv file from GAD (https://geneticassociationdb.nih.gov, the data was frozen as of 09/01/2014). Gene-disease associations in GAD used MeSH descriptors for diseases. After parsing the field “MESHDIS”, we obtained the curated gene-disease associations from GAD. We retrieved gene-disease and drug/chemical-disease associations from the CTD (http://ctd.mdibl.org/downloads/) on 11/17/2016. Among the 29,645 curated gene-disease associations in CTD, the vast majority used MeSH descriptors for diseases, but 5.16% used OMIM descriptors. To facilitate the integration with GAD data, we only retained CTD gene-disease associations using MeSH descriptors for diseases. According to the “Direct Evidence” code, these associations were divided into two parts: CTD_curated and CTD_inferred. The chemical-disease associations marked as “therapeutic” in CTD_curated were used as the gold standard for drug-disease associations. For gene-disease associations, we used the intersection or union of CTD_curated and GAD to define gold standards with different levels of stringency.

For performance comparison, we included CTD_inferred as described above and COREMINE. The disease names used by COREMINE were mapped to MeSH terms by “MeSH Browser” [16] or “MeSH ON DEMAND” [17] developed by NLM. The “MeSH Browser” can directly identify the alias of a MeSH term and the mapping by “MeSH ON DEMAND” is supervised by biologists.

To evaluate the retrieval performance, we used several metrics including precision, recall, F-measure, and Mean Average Precision (MAP). F-measure is calculated as 2*pr*/(*p* + *r*), where *p* represents the precision defined as |{*relevant diseases*} ∩ {*retrieved diseases*}|/|{*retrieved diseases*}| and *r* represents the recall defined as |{*relevant diseases*} ∩ {*retrieved diseases*}|/|{ *relevant diseases*}|. To measure precision at a fixed low level of retrieved results, we calculated precision at the top *k* retrieved diseases, where *k* = 10, 50 and 100. The MAP score for a set of queries is the mean of the average precision scores for each query:

$$ \mathrm{MAP}=\frac{1}{\mathrm{Q}}\sum \limits_{q=1}^{\mathrm{Q}}\mathrm{AveP}\left(\mathrm{q}\right) $$

where Q is the number of queries, and AveP(q) is the average precision scores for query q.

## Results

DLAD4U user interface accepts any valid queries for PubMed, and the output results include a ranked disease list, information associated with each disease, chronologically-ordered supporting publications, a summary of the run, and links for file export (Additional file 1: Figure S1). Because the gene-disease associations and drug-disease associations are the best studied disease-related relationships, we used gene terms and drug/chemical terms as queries to evaluate the quality of the retrieved disease lists.

### Gene-disease association distribution

**Fig. 1:**
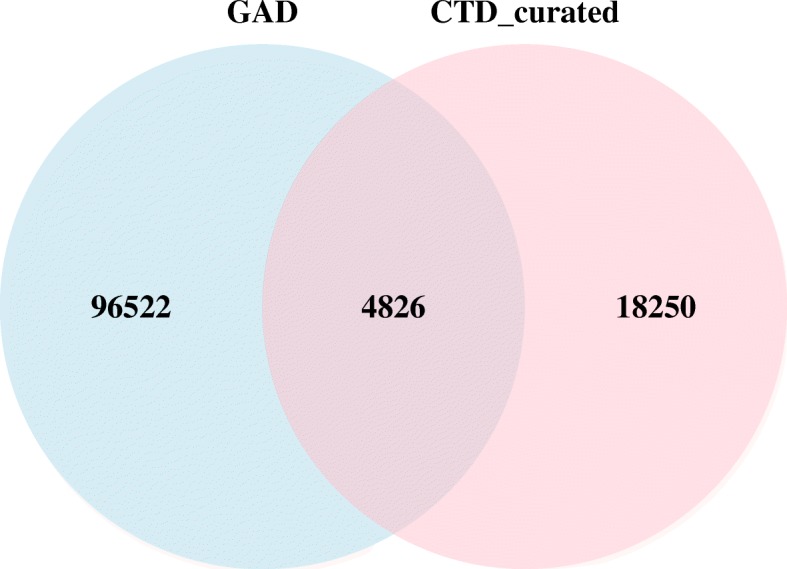
Venn diagram of data overlap between GAD and CTD_curated on gene-disease associations

**Fig. 2:**
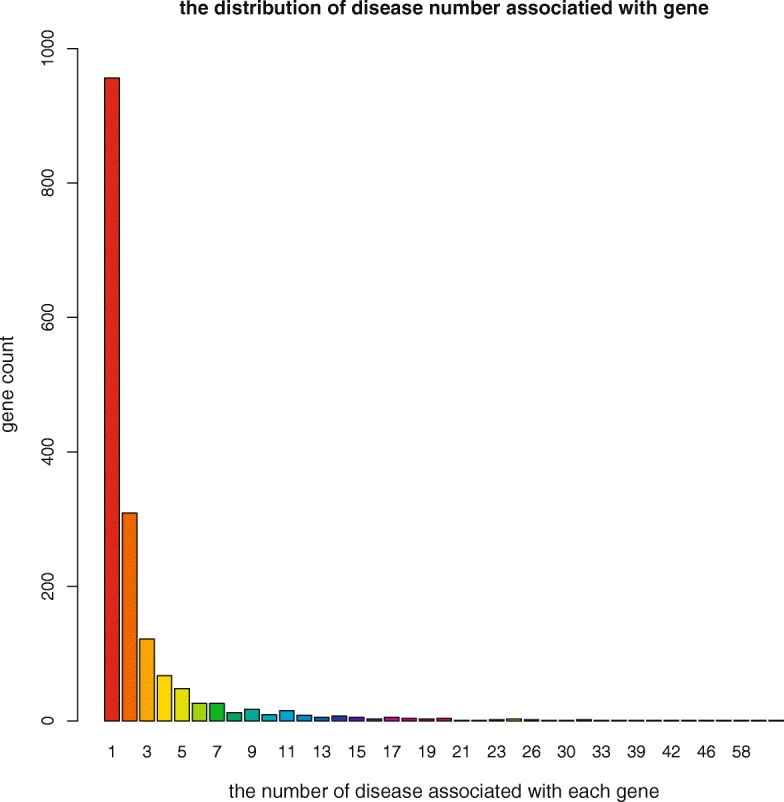
The distribution of the number of diseases associated with each gene. This is based on the gene-disease associations reported by both GAD and CTD_curated

The numbers of gene-disease associations curated by CTD_curated and GAD are shown in Fig. 1. The overlap between the two databases was less than 5% of the total associations. Among the gene-disease associations in the overlap of GAD and CTD_curated, we calculated the number of diseases associated with each gene, and 57% genes were associated with only one disease (Fig. 2). We labeled this subset of associations as one-to-one gene-disease associations and the others as one-to-many gene-disease associations. Because more than half of gene-disease associations were one-to-one associations, we focused on these associations first to evaluate the performance of DLAD4U. Performance on one-to-many gene-disease associations were evaluated separately.

### One-to-one gene-disease associations

For the one-to-one gene-disease associations, we used the 100 most confident gene-disease associations selected based on the number of supporting publications provided in the two databases as the gold standard for the evaluation. For the selected associations, the numbers of supporting publications ranged from 2 to 51 in CTD and 2 to 131 in GAD. We excluded associations supported by only one publication to avoid irreproducible gene-disease associations. We used the 100 genes in these selected associations as queries to evaluate the quality of retrieved disease lists.

We listed the ranks of the corresponding gold standard diseases in the disease lists retrieved by DLAD4U, COREMINE and CTD_inferred, respectively, for the 100 genes in Additional file 1: Table S1. The MAP scores and the statistics of the rank of corresponding gold standard diseases can be found in Table 1. Among the 100 genes, 67 disease lists returned by DLAD4U ranked the corresponding gold standard diseases at the first place, 77 among the top 2, and 91 among the top 5, which are all better than results from COREMINE and CTD_inferred. The higher ranks of gold standard diseases led to an MAP score of 0.77 for DLAD4U, which is 22% higher than that for COREMINE (0.63) and an order of magnitude higher than that for CTD_inferred (0.08).

**Table 1:** Performance evaluation of disease lists retrieved for one-to-one gene-disease associations

	DLAD4U	COREMINE	CTD_inferred
MAP	0.77	0.63	0.08
Rank of gold standard at top 1	67	50	5
Rank of gold standard at top 2	77	64	7
Rank of gold standard at top 5	91	77	8

For queries where the corresponding gold standard disease did not rank among the top 5 by DLAD4U, Additional file 1: Table S2 shows the top disease along with the first 10 supporting publications returned by DLAD4U. We found strong evidence supporting the relationships between these non-gold standard diseases and corresponding query genes. For example, DLAD4U linked the APOB gene to coronary disease with 819 supporting publications and a score of 1819. The APOB gene encodes the apolipoprotein B (apoB) protein, which is an important component of many lipoproteins that are involved in cardiovascular disease. It has been shown that the apoB/apoA-I ratio is superior to any of the cholesterol ratios in predicting the risk of coronary disease [18]. As another example, DLAD4U linked the BCHE gene to Alzheimer’s disease with 153 supporting publications and a score of 380. A meta-analysis based on 56 genetic case-control studies of 12,563 cases and 12,622 controls associated the BCHE gene with Alzheimer’s disease [19].

### One-to-many gene-disease associations

For one-to-many gene-disease associations, we used the top 10 genes ranked by the count of associated diseases from the overlap of GAD and CTD_curated as queries to evaluate the performance of DLAD4U, COREMINE and CTD_inferred. For the gold standard, we used the following four criteria:

1. the union of gene-disease associations in CTD_curated and GAD;
2. the union of gene-disease associations in CTD_curated and GAD with more than 1 supporting publication in each database;
3. the intersection gene-disease associations in CTD_curated and GAD;
4. the intersection gene-disease associations in CTD_curated and GAD with more than 1 supporting publication in each database.

For each query, using corresponding disease lists created based on the above mentioned four criteria as gold standards, we calculated precision, recall and F-measure of the top 100 retrieved diseases returned by DLAD4U, COREMINE, and CTD_inferred.

The precision, recall and F-measure of each query are listed in Additional file 1: Table S3, and the mean values are listed in Table 2. The mean precision, recall and F-measure scores of DLAD4U are all higher than those of COREMINE and CTD_inferred for all four criteria. The highest mean precision is 0.85 ± 0.10 on criterion 1, the highest mean recall is 0.86 ± 0.12 on criterion 4 and the highest F-measure is 0.49 ± 0.08 on criterion 2. With the stringency increased from criterion 1 to criterion 4, the mean recall increased from 0.27 ± 0.09 to 0.86 ± 0.12, while the mean precision decreased from 0.85 ± 0.10 to 0.13 ± 0.05 for DLAD4U. Results from COREMINE and CTD_inferred showed a similar trend. Both DLAD4U and COREMINE performed much better than CTD_inferred, consistent with our observations in the study of one-to-one gene-disease associations.

**Table 2:** Overall performance evaluation of disease lists retrieved for one-to-many gene-disease associations

	Criterion 1			Criterion 2			Criterion 3			Criterion 4		
	P	R	F	P	R	F	P	R	F	P	R	F
DLAD4U	0.85	0.27	0.39	0.72	0.40	0.49	0.30	0.61	0.40	0.13	0.86	0.22
COREMINE	0.70	0.22	0.32	0.56	0.32	0.39	0.25	0.49	0.32	0.1	0.69	0.18
CTD_inferred	0.53	0.16	0.24	0.36	0.19	0.24	0.18	0.36	0.23	0.08	0.54	0.13

*P* Precision, *R* Recall, *F* F-measure

**Fig. 3:**
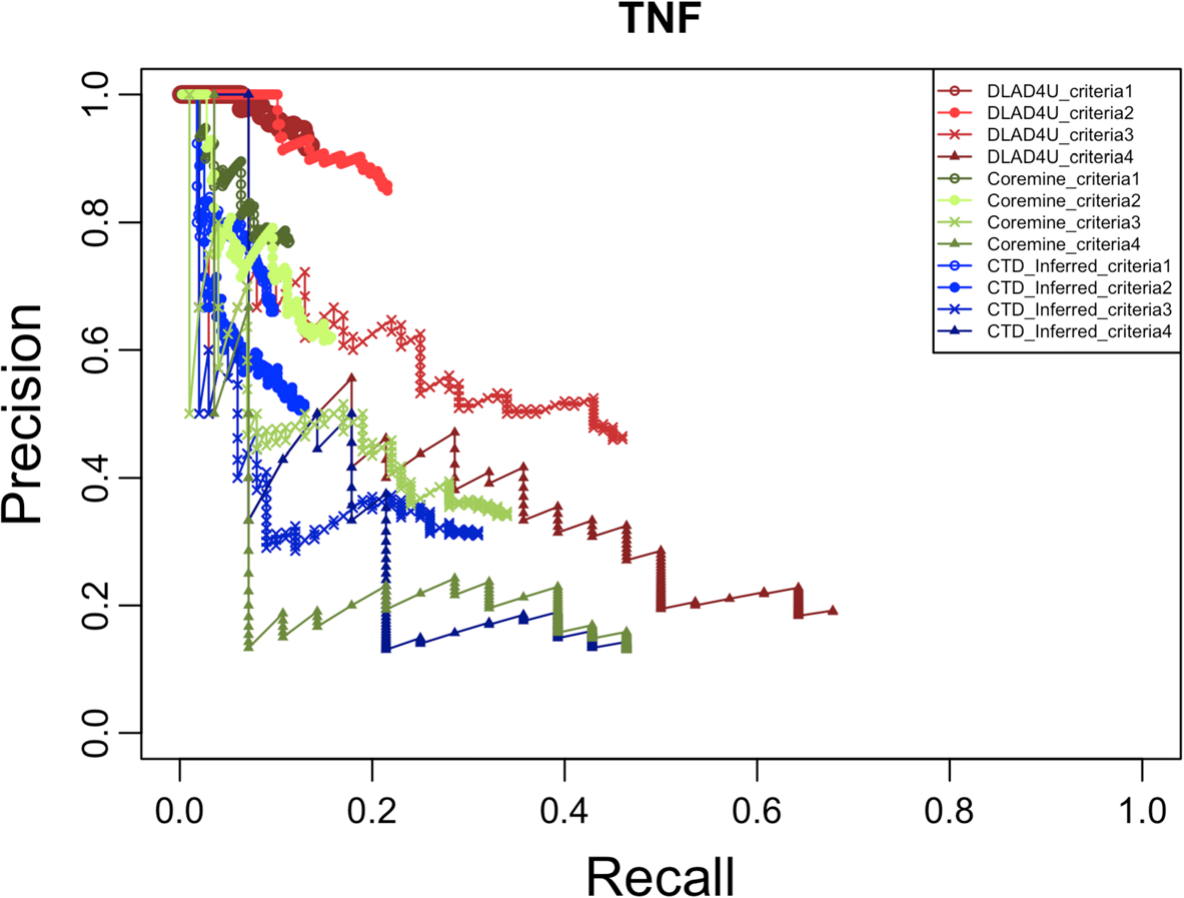
Precision/recall curves for TNF. Precision/recall curves for DLAD4U, COREMINE and CTD_inferred are colored in red, green and blue respectively. Different patterns are used to distinguish different criteria

**Fig. 4:**
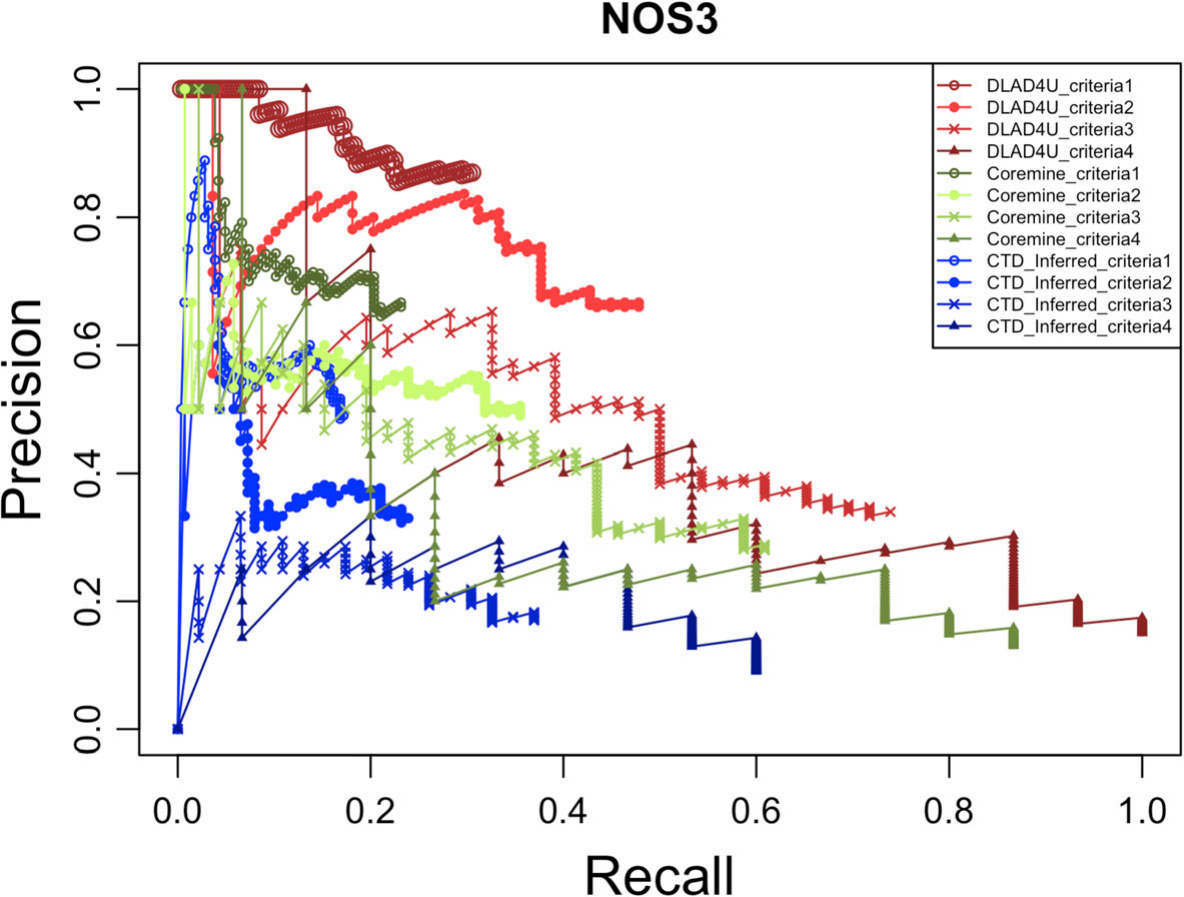
Precision/recall curves for NOS3. Precision/recall curves for DLAD4U, COREMINE and CTD_inferred are colored in red, green and blue respectively. Different patterns are used to distinguish different criteria

The precision/recall curves for the genes TNF and NOS3 are shown in Figs.3 and 4, respectively. Precision/recall curves for the other eight genes can be found in Additional file 1: Figures S2-S9. The precision/recall curve plot of TNF is a typical example of the 10 genes, in which DLAD4U clearly outperformed COREMINE and CTD_inferred (Fig. 3). However, DLAD4U did not always have the leading position in all conditions. For example, COREMINE had the leading position with criterion 2 and 3 in the low recall zone for NOS3, but the advantage disappeared with the increase of recall (Fig. 4).

For ranked disease lists in a web-based application, the number of relevant diseases on the first page is a major consideration. To measure precision at a fixed low level of retrieved results, e.g., the top 10 results, “Precision at K” is usually used. For this purpose, precisions for the top 10 (k = 10), 50 (k = 50) and 100 (k = 100) diseases were calculated for DLAD4U, COREMINE and CTD_inferred using all 4 criteria.

All precision values at the top 10, 50 and 100 diseases for each query are listed in Additional file 1: Table S4, and the corresponding mean precision values are listed in Table 3. Overall, DLAD4U maintained the highest precision at the top 10, 50 and 100 diseases compared to COREMINE and CTD_inferred based on all 4 different gold standards. For a few criteria and gene combinations, COREMINE had a higher precision at the top 10 (NOS3 on criterion 2 and 3, PTGS2 on criterion 3), however, the advantage disappeared at the top 50 and 100 (Additional file 1: Table S4). The highest mean precision at the top 10, 50 and 100 occurred with criterion 1 (0.98 ± 0.06, 0.92 ± 0.08 and 0.85 ± 0.10) due to the relatively loose stringency used in criterion 1. COREMINE and CTD_inferred also had the best performance with criterion 1 compared to other criteria.

**Table 3:** Average precision at top k for disease lists retrieved for one-to-many gene-disease associations

	Criterion 1			Criterion 2			Criterion 3			Criterion 4		
	P@	P@	P@	P@	P@	P@	P@	P@	P@	P@	P@	P@
	10	50	100	10	50	100	10	50	100	10	50	100
DLAD4U	0.98	0.92	0.85	0.90	0.82	0.72	0.63	0.43	0.30	0.46	0.22	0.13
COREMINE	0.89	0.77	0.70	0.73	0.66	0.56	0.49	0.33	0.25	0.28	0.16	0.10
CTD_inferred	0.74	0.61	0.53	0.55	0.41	0.36	0.28	0.22	0.18	0.20	0.10	0.08

*P* : Precision

### Drug/chemical-disease associations

The performance of DLAD4U was further evaluated by drug/chemical-disease associations. The human curated drug/chemical-disease associations in CTD_curated were used as the gold standard. We used the top 100 drug/chemical terms ranked by the count of supporting publications as queries and the corresponding disease as the gold standard to evaluate the quality of the retrieved disease lists.

For each drug/chemical query, the rank of the corresponding gold standard diseases in the disease list returned by DLAD4U, COREMINE, and CTD_inferred is listed in Additional file 1: Table S5. Table 4 lists the MAP scores and rank statistics of corresponding gold standard diseases for 100 query drugs. The MAP scores of DLAD4U (0.90) are higher than that of COREMINE (0.77) and much higher than that of CTD_inferred (0.15).

**Table 4:** Performance evaluation of disease lists retrieved for drug/chemical-disease associations

	DLAD4U	COREMINE	CTD_inferred
MAP	0.90	0.77	0.15
Rank of gold standard at top 1	69	56	5
Rank of gold standard at top 2	86	74	11
Rank of gold standard at top 5	97	91	22

Among the 100 drug/chemical queries, 82 disease lists returned by DLAD4U ranked the corresponding gold standard diseases at the first place, 90 among the top 2, and 97 among the top 5, which are all better than results from COREMINE and CTD_inferred. The 3 drug-disease associations which were not in the top 5 diseases returned by DLAD4U are “Methotrexate” and “Osteosarcoma”, “Propranolol” and “Tachycardia” and “Vancomycin” and “Endocarditis, Bacterial” (Additional file 1: Table S5). We noticed that all these drugs are associated with multiple diseases in the gold standard, and the best diseases returned for these drugs were also in the gold standard.

## Discussion

DLAD4U showed better performance in discovering gene-disease associations and drug/chemical-disease associations compared with COREMINE and CTD_inferred. COREMINE is a concept-oriented application for mining existing biomedical literature to build disease lists [10]. The application attempts to build the relationship between publications and concepts (including diseases) through text mining tools. CTD_inferred predicts new relationships using a “guilt-by-association” approach as described in the background section. DLAD4U is built upon curated publication-MeSH mapping available from NCBI. Our results underscore the high quality of this NCBI resource and the limitation of existing computational approaches, especially the “guilt-by-association” approach that showed the poorest performance.

We noticed two main reasons for the false-positive diseases retrieved by DLAD4U. One is the ambiguity of the query term. For example, “REN” is the gene symbol of the “renin” gene, and it is also the abbreviation of the organ “renal”. Furthermore, there is a medical journal name abbreviated “Ren Fail” (Full name is “Renal Failure”). When REN is queried, DLAD4U understands it as both “renin” and “renal”, and returns the related disease list. Another reason for the false-positives is the incompleteness of the gold standard. The manually curated gold standard is hard to keep up with the explosive growth of biomedical publications. Furthermore, the stringent criteria we used for the gold standard might also lead to incompleteness. For example, for the gene “CYP1A1”, the associated disease in the gold standard is “Prostatic Neoplasms” which is supported by 38 publications in GAD and 2 publications in CTD_curated. The query for “CYP1A1” in DLAD4U identified “Lung Neoplasms” as the best disease. Although this relationship is supported by 115 publications in GAD, it is not included in the CTD-curated, and thus is not included in our gold standard.

A notable feature of DLAD4U is its flexibility and user-friendliness. Because the search engine is powered by PubMed’s API, and the application behaves similarly to PubMed searches. Although we only used genes and drugs for performance evaluation, the DLAD4U user interface accepts any valid queries for PubMed, such as proteins, pathways, biological processes, environment factors, life style factors, phenotypes, etc. Diseases can also be used as query terms to find other related diseases. DLAD4U can screen and identify related publications, retrieve relevant disease information, rank these diseases and finally send the result back to users. The output of DLAD4U is a simple list of diseases relevant to the query term along with supporting publications. In addition, DLAD4U is almost maintenance-free by using PubMed’s API. With frequent publication-to-disease link table update, which is automatable, DLAD4U would be up-to-date with current literature because queries are performed directly against the MEDLINE library.

Because of its simplicity and flexibility, DLAD4U has broad applications in biomedical research. For example, colorectal cancer researchers interested in microsatellite instability (MSI), a hypermutable phenotype with known clinical relevance in colorectal cancer, may use DLAD4U to identify additional cancer types with the same MSI phenotype to perform a multi-cancer study on this important phenotype. A query in DLAD4U returned colorectal cancer as the top hit, and other top-ranking cancers included stomach cancer, endometrial cancer, and breast cancer. Interestingly, a recent pan-cancer study of MSI based on whole-exome data from The Cancer Genome Atlas (TCGA) project also identified these four cancer types as the most MSI-prone among all 20 tumor types studied [20]. As another example, querying DLAD4U using Abemaciclib, an FDA-approved drug for hormone receptor–positive, human epidermal growth factor receptor 2–negative advanced or metastatic breast cancer, revealed its potential effectiveness in glioblastoma [21]. These examples demonstrate that DLAD4U can facilitate the design of studies on multiple diseases with shared molecular or clinical phenotypes as well as drug repurposing studies.

## Conclusions

We have developed DLAD4U, a new, user-friendly disease search engine. DLAD4U takes advantage of existing resources at NCBI to provide computational efficiency and uses statistical analyses to achieve high accuracy.

## Additional file

Additional file 1: This archive contains the additional figures and tables for DLAD4U: driving and prioritizing disease lists from PubMed Literature. **Table S1.** The Rank of corresponding good standard in disease lists for one-to-one gene-disease associations. **Table S2.** Top 1 disease retrieved by DLAD4U and not listed in gold standard. **Table S3.** Overall quality of the retrieved disease lists for one-to-many gene-disease associations. **Table S4.** Comparison of retrieved disease lists by precision at top k for one-to-many gene-disease associations. **Table S5.** The Rank of corresponding good standard drug in the disease lists. **Figure S1.** DLAD4U interface. **Figure S2.** Precision/recall curves for MTHFR gene. **Figure S3.** Precision/recall curves for IL6 gene. **Figure S4.** Precision/recall curves for TNF gene. **Figure S5.** Precision/recall curves for TGFB1 gene. **Figure S6.** Precision/recall curves for ACE gene. **Figure S7.** Precision/recall curves for PTGS2 gene. **Figure S8.** Precision/recall curves for SOD2 gene. **Figure S9.** Precision/recall curves for IL1B gene. (PDF 5291 kb)

## Abbreviations

BCHE: Butyrylcholinesterase
BeFree: Bio-Entity finder and relation extraction
CTD: Comparative toxicogenomics database
DLAD4U: Disease list automatically derived for you
GAD: Genetic association database
GLAD4U: Gene list automatically derived for you
GWAS: Genome-wide association studies
HTML: Hypertext markup language
LHGDN: Literature-derived human gene-disease network
MAP: Mean average precision
MeSH: Medical subject headings
MTHFR: Methylenetetrahydrofolate reductase
NCBI: National Center for biotechnology information
NOS3: Nitric oxide synthase 3
OMIM: Online mendelian inheritance in man
PheWAS: Phenome-wide association studies
PHP: Hypertext preprocessor
PMIDs: PubMed IDs
PTGS2: Prostaglandin-endoperoxide synthase 2
TNF: Tumor necrosis factor
XML: eXtensible markup language
