# Supplementary material for: DLAD4U: deriving and prioritizing disease lists from PubMed literature
Source: BMC Bioinformatics. 2018 Dec 28;19(Suppl 17):495. doi: 10.1186/s12859-018-2463-0 (PMC6309061; doi:10.1186/s12859-018-2463-0)
Supplement: Supplementary file 1 — This archive contains the additional figures and tables for DLAD4U: driving and prioritizing disease lists from PubMed Literature. Table S1. The Rank of corresponding good standard in disease lists for one-to-one gene-disease associations. Table S2. Top 1 disease retrieved by DLAD4U and not listed in gold standard. Table S3. Overall quality of the retrieved disease lists for one-to-many gene-disease associations. Table S4. Comparison of retrieved disease lists by precision at top k for one-to-many gene-disease associations. Table S5. The Rank of corresponding good standard drug in the disease lists. Figure S1. DLAD4U interface. Figure S2. Precision/recall curves for MTHFR gene. Figure S3. Precision/recall curves for IL6 gene. Figure S4. Precision/recall curves for TNF gene. Figure S5. Precision/recall curves for TGFB1 gene. Figure S6. Precision/recall curves for ACE gene. Figure S7. Precision/recall curves for PTGS2 gene. Figure S8. Precision/recall curves for SOD2 gene. Figure S9. Precision/recall curves for IL1B gene. (PDF 5291 kb) [file 12859_2018_2463_MOESM1_ESM.pdf]

Additional file 1: Figures and Tables for:
DLAD4U: driving and prioritizing disease lists from PubMed Literature

Table of Contents

Tables

Table S1. The Rank of corresponding good standard in disease lists for one-to-one gene-disease associations..2

Table S2. Top 1 disease retrieved by DLAD4U and not listed in gold standard ……........................... 5

Table S3. Overall quality of the retrieved disease lists for one-to-many gene-disease associations …................. 6

Table S4. Comparison of retrieved disease lists by precision at top k for one-to-many gene-disease associations..7

Table S5. The Rank of corresponding good standard drug in the disease lists 8

Figures

Figure S1. DLAD4U interface 11

Figure S2. Precision/recall curves for MTHFR gene 12

Figure S3. Precision/recall curves for IL6 gene 13

Figure S4. Precision/recall curves for TNF gene 14

Figure S5. Precision/recall curves for TGFB1 gene 15

Figure S6. Precision/recall curves for ACE gene 16

Figure S7. Precision/recall curves for PTGS2 gene 17

Figure S8. Precision/recall curves for SOD2 gene 18

Figure S9. Precision/recall curves for IL1B gene 19

Table S1. The Rank of corresponding good standard in disease lists for one-to-one gene-disease associations

Query (gene term)	Gold standard (disease)	rank at DLAD4U	rank at COREMINE	rank at CTD_inferred
ATP7B	Hepatolenticular Degeneration	1	1	73
APP	Alzheimer Disease	1	1	13
BCHE	Apnea	8	6	54
SOD1	Amyotrophic Lateral Sclerosis	1	1	340
CFH	Macular Degeneration	1	2	1338
SLC30A8	Diabetes Mellitus, Type 2	1	3	40
CDKAL1	Diabetes Mellitus, Type 2	1	2	69
KCNJ11	Diabetes Mellitus, Type 2	2	5	74
PSEN1	Alzheimer Disease	1	1	75
REN	Hypertension	3	1	1
MEFV	Familial Mediterranean Fever	1	1	0*
MSH2	Colorectal Neoplasms, Hereditary Nonpolyposis	1	1	0*
IRS1	Diabetes Mellitus, Type 2	2	2	83
GCK	Diabetes Mellitus, Type 2	1	1	139
KLK3	Prostatic Neoplasms	1	1	1
SRD5A2	Prostatic Neoplasms	1	7	22
CYP17A1	Prostatic Neoplasms	2	6	36
CHEK2	Breast Neoplasms	1	6	13
HLA-C	Psoriasis	1	1	381
ADD1	Hypertension	1	1	38
ARMS2	Macular Degeneration	1	1	0*
A2M	Alzheimer Disease	1	7	544
PARK7	Parkinson Disease	1	1	55
ATG16L1	Crohn Disease	1	1	296
HNF4A	Diabetes Mellitus, Type 2	1	1	63
PIK3CA	Breast Neoplasms	2	4	20
HTR2A	Schizophrenia	1	1	62
MTNR1B	Diabetes Mellitus, Type 2	1	2	219
BMPR2	Hypertension, Pulmonary	1	1	24
CYP21A2	Adrenal Hyperplasia, Congenital	1	1	0*
DHCR7	Smith-Lemli-Opitz Syndrome	1	1	785
VHL	Carcinoma, Renal Cell	3	3	346
NRG1	Schizophrenia	1	1	462
HTT	Huntington Disease	1	1	105
DRD4	Attention Deficit Disorder with Hyperactivity	1	1	121
MSMB	Prostatic Neoplasms	1	1	9
MSH6	Colorectal Neoplasms, Hereditary Nonpolyposis	1	1	0*
CYP1B1	Breast Neoplasms	1	4	17

FXN	Friedreich Ataxia	1	1	9
MC1R	Melanoma	1	1	60
RHO	Retinitis Pigmentosa	34	1	99
IRGM	Crohn Disease	1	1	780
FBN1	Marfan Syndrome	1	1	0*
PPARGC1A	Diabetes Mellitus, Type 2	3	2	102
UCP2	Diabetes Mellitus, Type 2	2	3	183
CYP1A1	Prostatic Neoplasms	11	49	88
C3	Macular Degeneration	44	6	1833
ABCC6	Pseudoxanthoma Elasticum	1	1	1549
TRAF1	Arthritis, Rheumatoid	1	4	305
HNF1B	Prostatic Neoplasms	8	48	305
SMN1	Muscular Atrophy, Spinal	1	1	0*
TLR4	Inflammation	1	1	1
ERCC2	Urinary Bladder Neoplasms	10	11	18
CLU	Alzheimer Disease	1	4	235
IRF6	Cleft Lip	2	1	88
PTPN11	Noonan Syndrome	1	1	0**
TARDBP	Amyotrophic Lateral Sclerosis	1	3	998
RNR1	Deafness	3	0**	1
F8	Hemophilia A	1	1	0*
ADAMTS9	Diabetes Mellitus, Type 2	1	2	347
LPL	Hypertriglyceridemia	2	2	18
LIPC	Diabetes Mellitus, Type 2	4	25	64
PDCD1	Lupus Erythematosus, Systemic	5	19	0
IL7R	Multiple Sclerosis	1	1	1354
IL1A	Inflammation	1	1	2
PSEN2	Alzheimer Disease	1	1	187
IGFBP3	Prostatic Neoplasms	3	6	41
RNASEL	Prostatic Neoplasms	1	2	78
CST3	Alzheimer Disease	5	29	303
ERCC6	Cockayne Syndrome	1	1	122
CHRNA5	Tobacco Use Disorder	1	1	88
APOB	Cardiovascular Diseases	6	13	18
CCL21	Arthritis, Rheumatoid	4	7	383
FGFR3	Urinary Bladder Neoplasms	1	6	163
NRAS	Melanoma	1	3	167
ITGAM	Lupus Erythematosus, Systemic	10	60	193
KCNE1	Long QT Syndrome	1	2	179
SLCO1B1	Muscular Diseases	1	1	15
GAA	Glycogen Storage Disease Type II	1	1	0*
PKD1	Polycystic Kidney, Autosomal Dominant	1	1	896
TNFSF4	Lupus Erythematosus, Systemic	1	2	188
CEBPA	Leukemia, Myeloid, Acute	1	1	269
CYP2D6	Parkinson Disease	5	10	179
TFR2	Hemochromatosis	1	1	663
CLPTM1L	Lung Neoplasms	1	2	156
PRKCQ	Arthritis, Rheumatoid	3	10	248

ATXN1	Spinocerebellar Ataxias	1	1	0*
IL1RL1	Asthma	2	3	56
ERG	Prostatic Neoplasms	2	2	0*
DRD3	Cocaine-Related Disorders	5	6	2
PICALM	Alzheimer Disease	1	1	157
MSX1	Cleft Palate	2	2	81
IDUA	Mucopolysaccharidosis I	1	1	0*
MPO	Inflammation	1	3	1
SIRT1	Insulin Resistance	3	2	48
INSR	Insulin Resistance	1	1	4
SLC2A9	Gout	1	3	51
BARD1	Neuroblastoma	3	9	265
KCNE2	Long QT Syndrome	1	1	131
REL	Arthritis, Rheumatoid	14	56	0*

*. Corresponding gold standard was not found in the disease list retrieved by CTD_inferred.

**. Corresponding gold standard was not found in the top 100 disease list retrieved by COREMINE.

Table S2. Top 1 disease retrieved by DLAD4U and not listed in gold standard

Gene	Top 1 disease	PMIDs*
APOB	Coronary Disease	26446158,26115072,26025389,25839937,25835441,25835440,25676271,25620253,25477346,25374385
BCHE	Alzheimer Disease	26978503,26363866,26254241,26090073,26057771,25868744,25425329,25345511,25301673,25240096
HNF1B	Diabetes Mellitus, Type 2	26340261,26329304,26287533,25741167,25700310,25581748,25414397,25367728,25324567,25136813
ERCC2	Lung Neoplasms	26731659,26426637,26253951,26159902,26001533,25744060,25069034,24933103,24845027,24716924
ITGAM	Inflammation	26731659,26426637,26253951,26159902,26001533,25744060,25069034,24933103,24845027,24716924
CYP1A1	Lung Neoplasms	26580399,26149476,25795230,25447411,25343551,25233467,25149827,25040976,24938875,24651647
REL	Cell Transformation, Neoplastic	25736765,25727407,25393674,23975431,21345476,20562914,19948376,19580428,19458071,19377508
RHO	Neoplasm Invasiveness	26671750,26642367,26505794,26446205,26426996,26424695,26371759,26370503,26319120,26258642
C3	Glomerulonephritis	26616334,26275270,26109684,26080801,25991042,25991041,25973075,25889427,25806731,25800445

Table S3. Overall quality of the retrieved disease lists for one-to-many gene-disease associations

Query		Criterion 1			Criterion 2			Criterion 3			Criterion 4		
		P	R	F	P	R	F	P	R	F	P	R	F
TNF	DLAD4U	0.92	0.14	0.24	0.85	0.22	0.34	0.46	0.46	0.46	0.19	0.68	0.30
	COREMINE	0.77	0.11	0.20	0.62	0.16	0.25	0.34	0.34	0.34	0.13	0.46	0.20
	CTD_Inferred	0.66	0.10	0.17	0.51	0.13	0.21	0.31	0.31	0.31	0.13	0.46	0.20
MTHFR	DLAD4U	0.98	0.21	0.34	0.93	0.33	0.49	0.29	0.48	0.36	0.08	0.73	0.14
	COREMINE	0.90	0.19	0.31	0.86	0.30	0.45	0.28	0.46	0.35	0.08	0.73	0.14
	CTD_Inferred	0.64	0.13	0.22	0.43	0.15	0.23	0.20	0.33	0.25	0.06	0.55	0.11
IL6	DLAD4U	0.93	0.19	0.31	0.85	0.35	0.49	0.34	0.59	0.43	0.10	0.77	0.18
	COREMINE	0.68	0.14	0.23	0.55	0.22	0.32	0.20	0.34	0.25	0.05	0.38	0.09
	CTD_Inferred	0.63	0.13	0.21	0.50	0.20	0.29	0.16	0.28	0.20	0.04	0.31	0.07
TP53	DLAD4U	0.91	0.30	0.45	0.80	0.50	0.61	0.34	0.72	0.46	0.19	0.95	0.32
	COREMINE	0.88	0.29	0.44	0.76	0.47	0.58	0.34	0.72	0.46	0.19	0.95	0.32
	CTD_Inferred	0.32	0.11	0.16	0.23	0.14	0.18	0.15	0.32	0.20	0.09	0.45	0.15
NOS3	DLAD4U	0.87	0.31	0.45	0.66	0.48	0.55	0.34	0.74	0.47	0.15	1.00	0.26
	COREMINE	0.66	0.23	0.34	0.49	0.36	0.41	0.28	0.61	0.38	0.13	0.87	0.23
	CTD_Inferred	0.49	0.17	0.25	0.33	0.24	0.28	0.17	0.37	0.23	0.09	0.60	0.16
TGFB1	DLAD4U	0.77	0.21	0.33	0.59	0.32	0.41	0.26	0.59	0.36	0.11	0.85	0.19
	COREMINE	0.57	0.15	0.24	0.43	0.23	0.30	0.20	0.45	0.28	0.10	0.77	0.18
	CTD_Inferred	0.50	0.13	0.21	0.28	0.15	0.20	0.15	0.34	0.21	0.09	0.69	0.16
ACE	DLAD4U	0.90	0.21	0.34	0.83	0.34	0.48	0.24	0.57	0.34	0.08	1.00	0.15
	COREMINE	0.77	0.18	0.29	0.67	0.27	0.39	0.21	0.50	0.30	0.07	0.88	0.13
	CTD_Inferred	0.69	0.16	0.26	0.55	0.22	0.32	0.21	0.50	0.30	0.07	0.88	0.13
PTGS2	DLAD4U	0.71	0.37	0.48	0.52	0.64	0.57	0.32	0.78	0.45	0.16	1.00	0.28
	COREMINE	0.58	0.30	0.40	0.41	0.51	0.45	0.26	0.63	0.37	0.13	0.81	0.22
	CTD_Inferred	0.42	0.22	0.29	0.21	0.26	0.23	0.15	0.37	0.21	0.09	0.56	0.16
SOD2	DLAD4U	0.67	0.31	0.42	0.45	0.50	0.47	0.28	0.72	0.40	0.14	0.74	0.24
	COREMINE	0.55	0.25	0.34	0.37	0.41	0.39	0.19	0.49	0.27	0.10	0.53	0.17
	CTD_Inferred	0.44	0.20	0.28	0.26	0.29	0.27	0.16	0.41	0.23	0.07	0.37	0.12
IL1B	DLAD4U	0.82	0.42	0.56	0.68	0.32	0.44	0.17	0.45	0.25	0.07	0.88	0.13
	COREMINE	0.66	0.34	0.45	0.48	0.23	0.31	0.15	0.39	0.22	0.04	0.50	0.07
	CTD_Inferred	0.53	0.27	0.36	0.25	0.12	0.16	0.13	0.34	0.19	0.04	0.50	0.07

P: Precision; R: Recall; F: F-measure

Table S4. Comparison of retrieved disease lists by precision at top k for multi gene-disease associations

		criterion 1			criterion 2			criterion 3			criterion 4		
		P@ 10	P@ 50	P@ 100	P@ 10	P@ 50	P@ 100	P@ 10	P@ 50	P@ 100	P@ 10	P@ 50	P@ 100
TNF	DLAD4U	1.00	0.98	0.92	1.00	0.92	0.85	0.70	0.56	0.46	0.50	0.28	0.19
	COREMINE	1.00	0.86	0.77	1.00	0.76	0.62	0.70	0.44	0.34	0.20	0.22	0.13
	CTD_Inferred	1.00	0.80	0.66	0.90	0.58	0.51	0.60	0.34	0.31	0.50	0.14	0.13
MTHFR	DLAD4U	1.00	0.98	0.98	1.00	0.96	0.93	0.80	0.42	0.29	0.40	0.16	0.08
	COREMINE	1.00	0.92	0.90	0.80	0.88	0.86	0.40	0.32	0.28	0.10	0.10	0.08
	CTD_Inferred	0.80	0.68	0.64	0.70	0.52	0.43	0.30	0.32	0.20	0.10	0.08	0.06
IL6	DLAD4U	1.00	0.96	0.93	1.00	0.84	0.85	0.60	0.44	0.34	0.50	0.16	0.10
	COREMINE	1.00	0.82	0.68	0.70	0.66	0.55	0.20	0.30	0.20	0.20	0.10	0.05
	CTD_Inferred	0.80	0.76	0.63	0.70	0.64	0.50	0.20	0.20	0.16	0.20	0.06	0.04
TP53	DLAD4U	1.00	0.98	0.91	1.00	0.94	0.80	0.60	0.50	0.34	0.50	0.32	0.19
	COREMINE	0.80	0.90	0.88	0.80	0.88	0.76	0.50	0.46	0.34	0.30	0.28	0.19
	CTD_Inferred	0.30	0.30	0.32	0.30	0.22	0.23	0.10	0.14	0.15	0.10	0.12	0.09
NOS3	DLAD4U	1.00	0.94	0.87	0.60	0.82	0.66	0.50	0.46	0.34	0.40	0.26	0.15
	COREMINE	1.00	0.70	0.66	0.70	0.54	0.49	0.60	0.40	0.28	0.40	0.22	0.13
	CTD_Inferred	0.80	0.56	0.49	0.60	0.36	0.33	0.30	0.24	0.17	0.30	0.16	0.09
TGFB1	DLAD4U	1.00	0.86	0.77	1.00	0.72	0.59	0.70	0.44	0.26	0.60	0.20	0.11
	COREMINE	0.90	0.64	0.57	0.60	0.52	0.43	0.60	0.26	0.20	0.50	0.16	0.10
	CTD_Inferred	0.70	0.64	0.50	0.50	0.36	0.28	0.40	0.20	0.15	0.40	0.12	0.09
ACE	DLAD4U	1.00	1.00	0.90	1.00	0.92	0.83	0.80	0.38	0.24	0.50	0.14	0.08
	COREMINE	1.00	0.88	0.77	1.00	0.80	0.67	0.70	0.32	0.21	0.30	0.12	0.07
	CTD_Inferred	0.80	0.82	0.69	0.80	0.70	0.55	0.20	0.26	0.21	0.10	0.12	0.07
PTGS2	DLAD4U	0.80	0.76	0.71	0.80	0.68	0.52	0.70	0.46	0.32	0.60	0.32	0.16
	COREMINE	0.80	0.66	0.58	0.70	0.56	0.41	0.80	0.40	0.26	0.50	0.24	0.13
	CTD_Inferred	0.60	0.48	0.42	0.30	0.24	0.21	0.30	0.16	0.15	0.10	0.06	0.09
SOD2	DLAD4U	1.00	0.82	0.67	0.70	0.62	0.45	0.40	0.36	0.28	0.30	0.20	0.14
	COREMINE	0.70	0.62	0.55	0.50	0.42	0.37	0.10	0.20	0.19	0.10	0.10	0.10
	CTD_Inferred	0.70	0.48	0.44	0.40	0.24	0.26	0.20	0.18	0.16	0.10	0.06	0.07
IL1B	DLAD4U	1.00	0.88	0.82	0.90	0.82	0.68	0.50	0.28	0.17	0.30	0.12	0.07
	COREMINE	0.70	0.68	0.66	0.50	0.56	0.48	0.30	0.16	0.15	0.20	0.06	0.04
	CTD_Inferred	0.90	0.58	0.53	0.30	0.24	0.25	0.20	0.12	0.13	0.10	0.04	0.04

P: Precision

Table S5. The Rank of corresponding good standard drug in the disease lists

Query (drug/chemical)	Gold standard (disease)	rank at DLAD4U	rank at COREMINE	rank at CTD_inferred
ABVD protocol	Hodgkin Disease	1	1	323
adefovir dipivoxil	Hepatitis B, Chronic	1	1	191
Albuterol	Asthma	1	1	5
Amiodarone	Arrhythmias, Cardiac	2	3	12
Amiodarone	Atrial Fibrillation	1	2	320
Amiodarone	Tachycardia, Ventricular	3	1	0
Amitriptyline	Depressive Disorder	1	1	0
Amlodipine	Hypertension	1	1	2
Angiotensin-Converting Enzyme Inhibitors	Hypertension	1	1	516
Angiotensin-Converting Enzyme Inhibitors	Heart Failure	2	1	1106
Antimony Sodium Gluconate	Leishmaniasis, Visceral	1	1	2
arsenic trioxide	Leukemia, Promyelocytic, Acute	1	1	50
Atenolol	Hypertension	1	1	0
Beclomethasone	Asthma	1	1	14
benazepril	Hypertension	1	1	1567
Bortezomib	Multiple Myeloma	1	1	0
Bupivacaine	Pain, Postoperative	1	0	1
Bupropion	Depressive Disorder	2	3	196
Carbamazepine	Epilepsy	1	1	25
Carbamazepine	Seizures	3	2	2
carbidopa, levodopa drug combination	Parkinson Disease	1	1	69
carvedilol	Heart Failure	1	1	3
Cisplatin	Carcinoma, Non-Small-Cell Lung	4	2	996
Cisplatin	Ovarian Neoplasms	3	1	28
Citalopram	Depressive Disorder	2	2	6
Clonidine	Hypertension	1	2	235
Clozapine	Schizophrenia	1	1	2
Cyclophosphamide	Breast Neoplasms	1	7	57
Cytarabine	Leukemia, Myeloid, Acute	1	1	155
Dacarbazine	Melanoma	2	5	1989
Dexamethasone	Multiple Myeloma	1	3	3
Diazepam	Seizures	1	2	45
Dizocilpine Maleate	Seizures	1	4	350
Doxorubicin	Breast Neoplasms	1	4	0
Enalapril	Hypertension	1	1	2
Epirubicin	Breast Neoplasms	1	1	1147
Fentanyl	Pain	2	4	233
Fluorouracil	Colorectal Neoplasms	3	1	413
Fluoxetine	Depressive Disorder	1	1	0
Haloperidol	Schizophrenia	1	2	1181

Hepatitis B Vaccines	Hepatitis B	1	1	296
Hydralazine	Hypertension	1	1	7
Hydrochlorothiazide	Hypertension	1	1	4
Imipramine	Depressive Disorder	1	1	37
Indapamide	Hypertension	1	1	6
Indinavir	HIV Infections	1	1	16
Isoniazid	Tuberculosis	2	1	921
Ketamine	Pain	1	20	53
Labetalol	Hypertension	1	1	125
Lamivudine	Hepatitis B	3	2	1
Lamivudine	Hepatitis B, Chronic	2	1	3
Levodopa	Parkinson Disease	1	1	14
Levodopa	Parkinsonian Disorders	3	3	3
Lidocaine	Pain	1	21	1
Lisinopril	Hypertension	1	1	27
Lithium	Bipolar Disorder	1	1	55
Lithium Carbonate	Bipolar Disorder	1	1	448
Losartan	Hypertension	1	1	45
Mesalamine	Colitis, Ulcerative	1	1	137
Methotrexate	Precursor Cell Lymphoblastic Leukemia-Lymphoma	4	2	196
Methotrexate	Arthritis, Rheumatoid	1	1	4
Methotrexate	Psoriasis	3	3	53
Methotrexate	Osteosarcoma	15	14	70
Methyldopa	Hypertension	1	1	10
Methylphenidate	Attention Deficit Disorder with Hyperactivity	1	1	129
Metoclopramide	Vomiting	1	0	4
Metoprolol	Hypertension	1	2	38
Morphine	Pain	1	7	82
Morphine	Pain, Postoperative	2	0	2
Nifedipine	Hypertension	1	2	10
Nifedipine	Angina Pectoris	2	3	0
olanzapine	Schizophrenia	1	1	1
Paclitaxel	Breast Neoplasms	1	5	179
Penicillamine	Arthritis, Rheumatoid	1	3	37
Penicillamine	Hepatolenticular Degeneration	2	1	44
Phenobarbital	Seizures	2	2	3
Phenytoin	Seizures	2	3	22
Phenytoin	Epilepsy	1	1	1
Prazosin	Hypertension	1	2	835
Propranolol	Hypertension	1	3	43
Propranolol	Tachycardia	7	1	6
resveratrol	Inflammation	2	2	151
Ribavirin	Hepatitis C, Chronic	1	1	4
Ribavirin	Hepatitis C	2	2	43
Rifampin	Tuberculosis	2	2	382
Risperidone	Schizophrenia	1	1	3894

Ritodrine	Obstetric Labor, Premature	1	1	8
Sumatriptan	Migraine Disorders	1	1	24
Tamoxifen	Breast Neoplasms	1	1	3
Thalidomide	Multiple Myeloma	1	1	72
Tretinoin	Leukemia, Promyelocytic, Acute	1	1	174
Valproic Acid	Seizures	3	3	6
Valproic Acid	Epilepsy	1	1	21
Valproic Acid	Bipolar Disorder	2	2	0
Valsartan	Hypertension	1	1	51
Vancomycin	Staphylococcal Infections	1	2	588
Vancomycin	Endocarditis, Bacterial	7	4	159
Verapamil	Hypertension	1	12	81
Vincristine	Precursor Cell Lymphoblastic Leukemia-Lymphoma	5	5	42
zafirlukast	Asthma	1	1	53

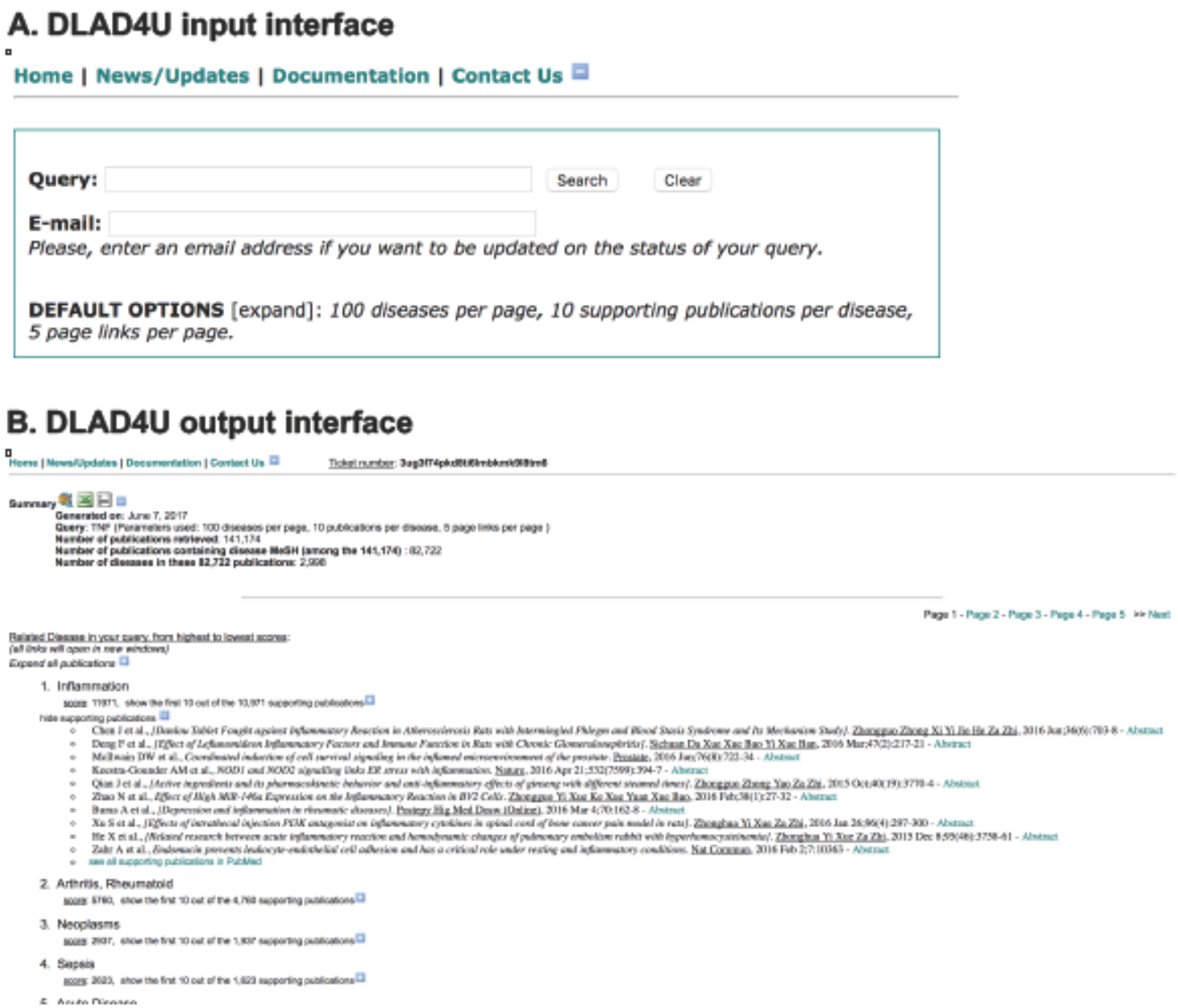

Figure S1. DLAD4U interface. (A) Input interface (B) Output interface.

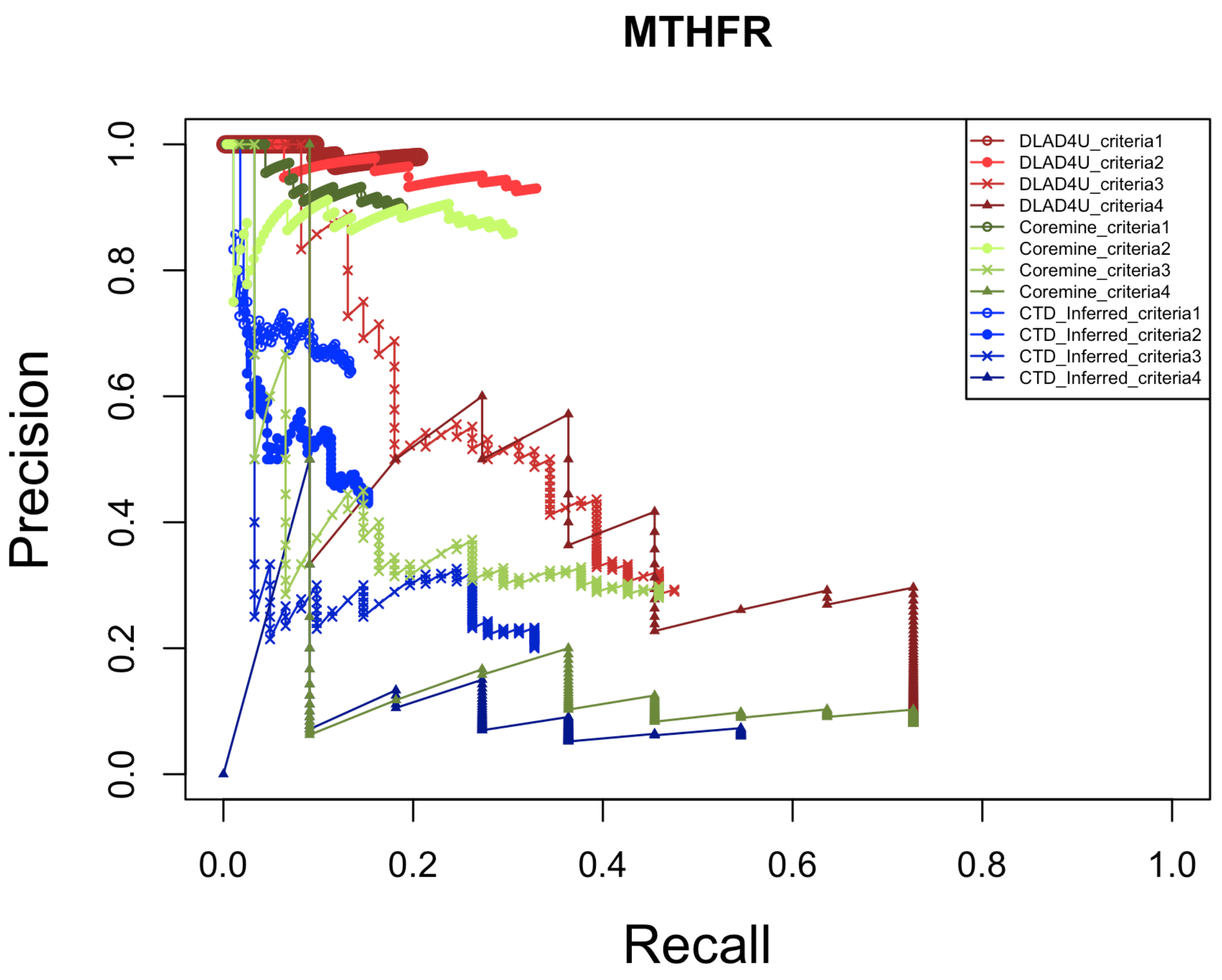

Figure S2. Precision/recall curves for MTHFR gene. Precision/recall curves for DLAD4U, COREMINE and CTD_inferred are colored in red, green and blue respectively. Different patterns are used to distinguish different criteria.

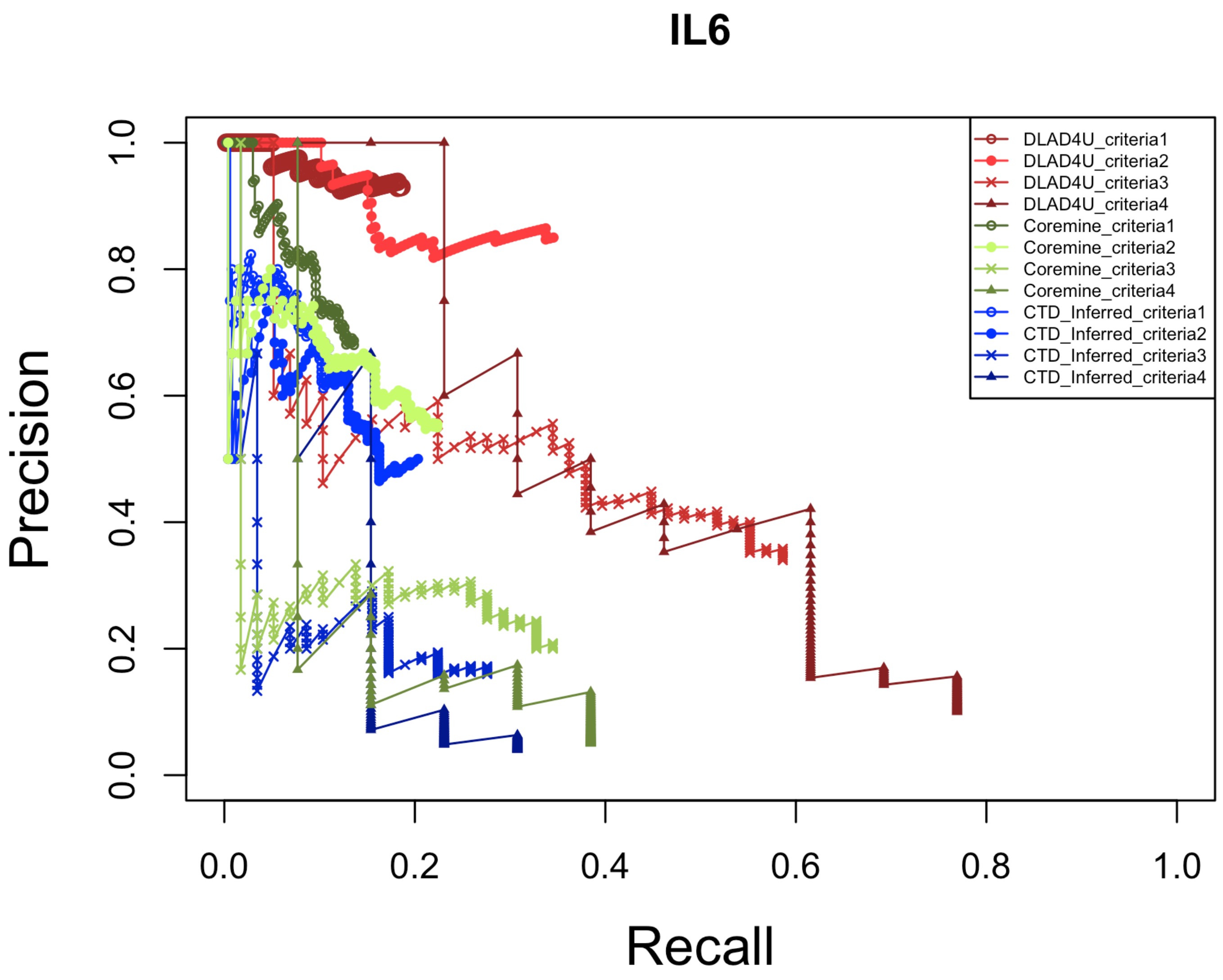

Figure S3. Precision/recall curves for IL6 gene. Precision/recall curves for DLAD4U, COREMINE and CTD_inferred are colored in red, green and blue respectively. Different patterns are used to distinguish different criteria.

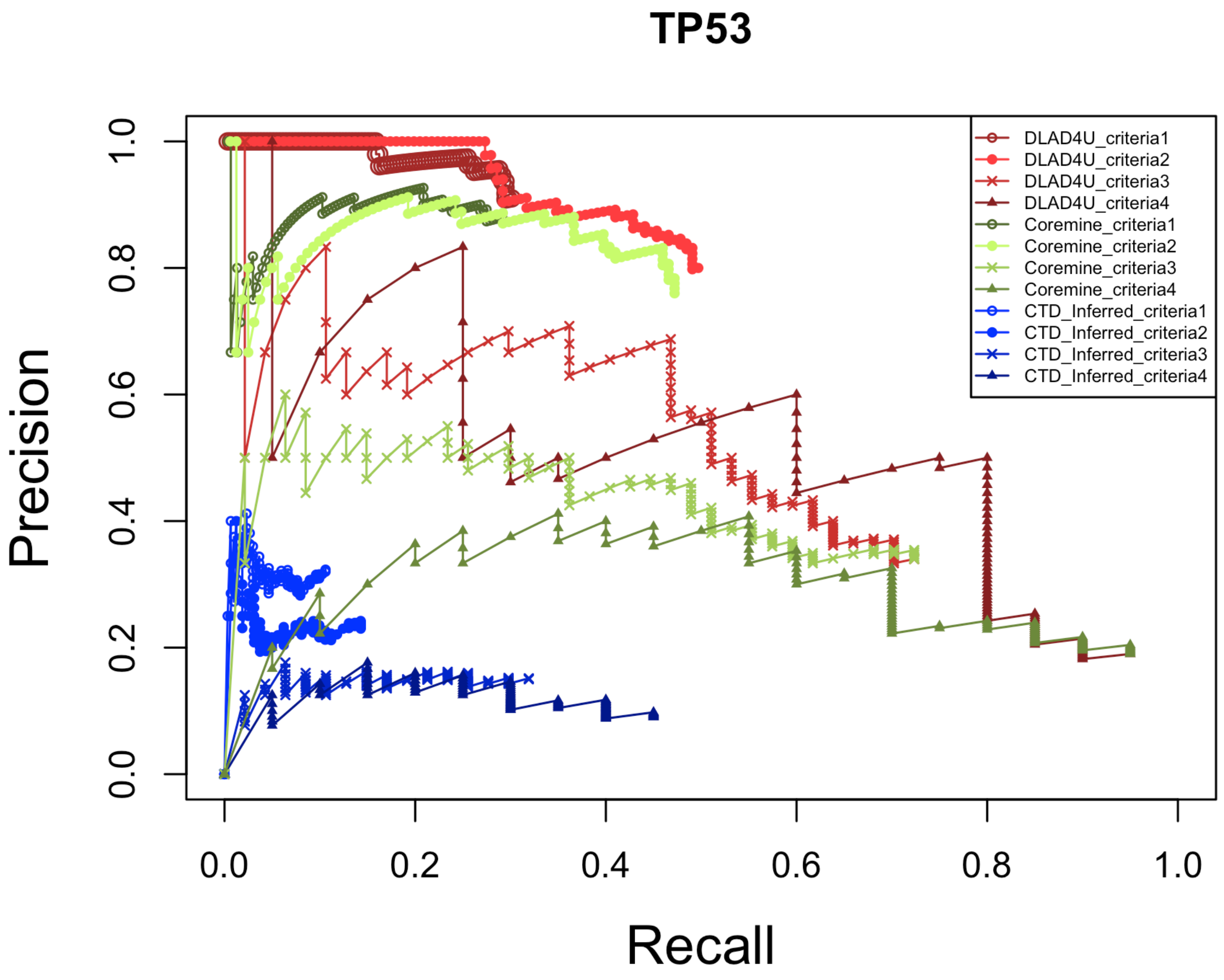

Figure S4. Precision/recall curves for TP53 gene. Precision/recall curves for DLAD4U, COREMINE and CTD_inferred are colored in red, green and blue respectively. Different patterns are used to distinguish different criteria.

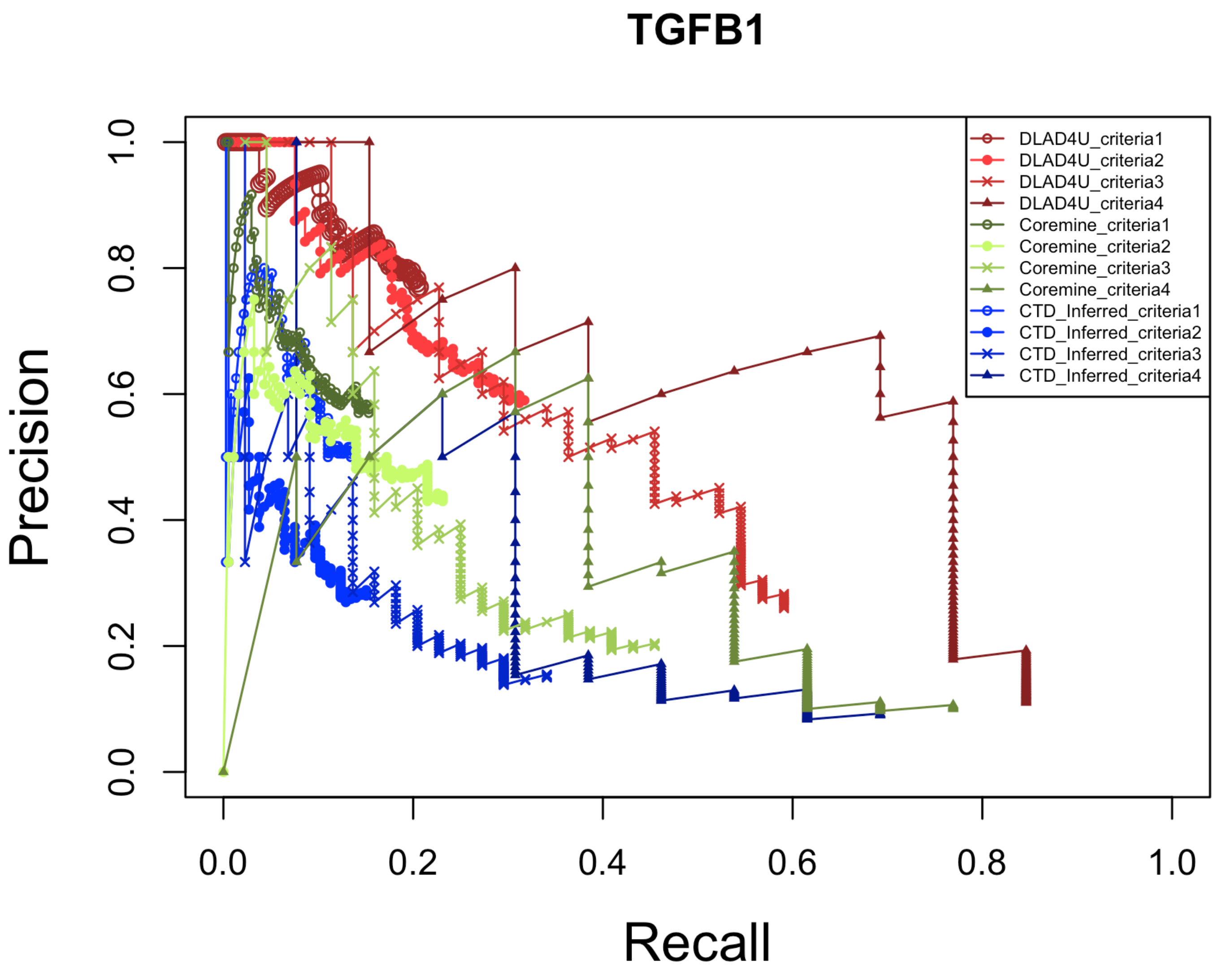

Figure S5. Precision/recall curves for TGFB1 gene. Precision/recall curves for DLAD4U, COREMINE and CTD_inferred are colored in red, green and blue respectively. Different patterns are used to distinguish different criteria.

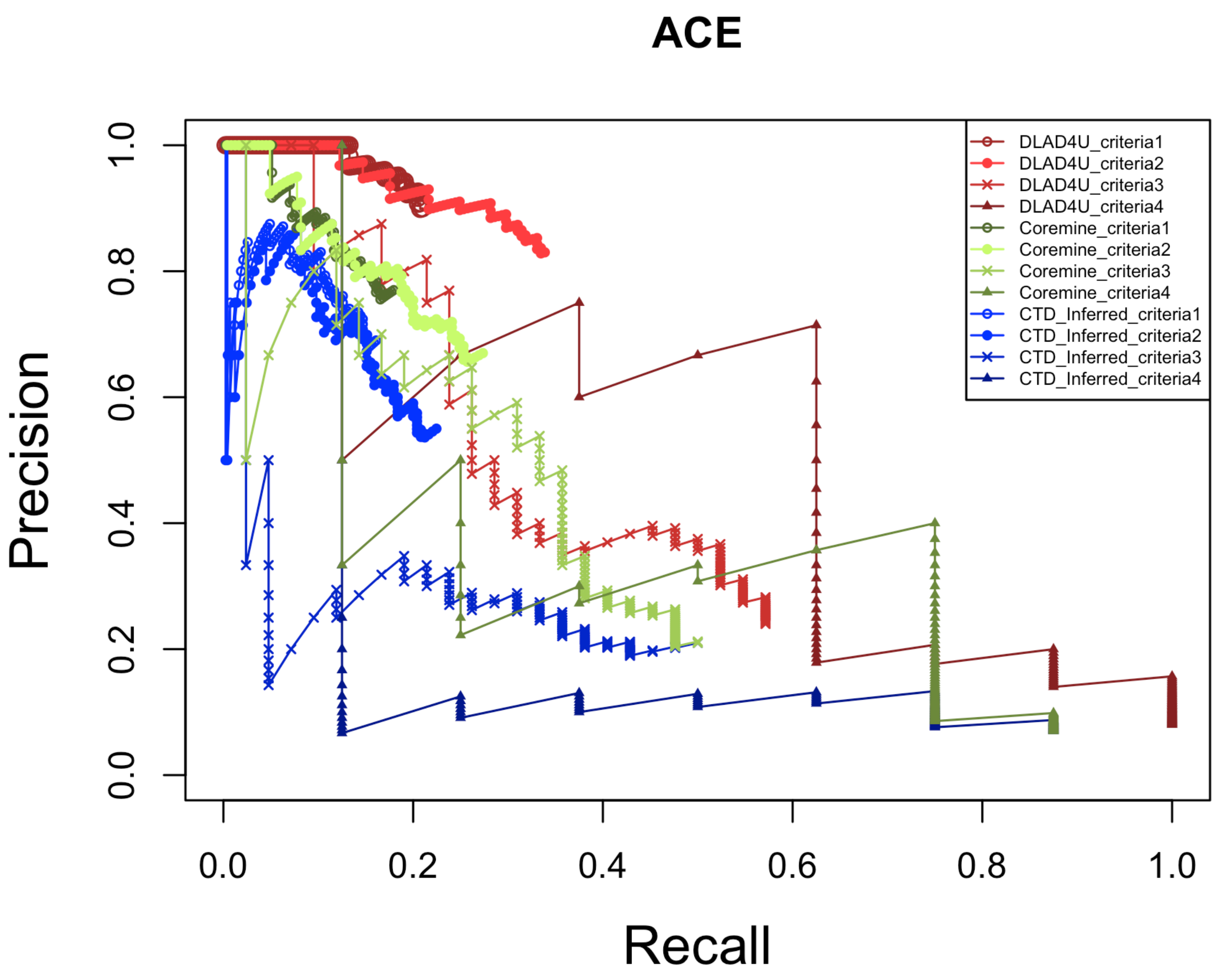

Figure S6. Precision/recall curves for ACE gene. Precision/recall curves for DLAD4U, COREMINE and CTD_inferred are colored in red, green and blue respectively. Different patterns are used to distinguish different criteria.

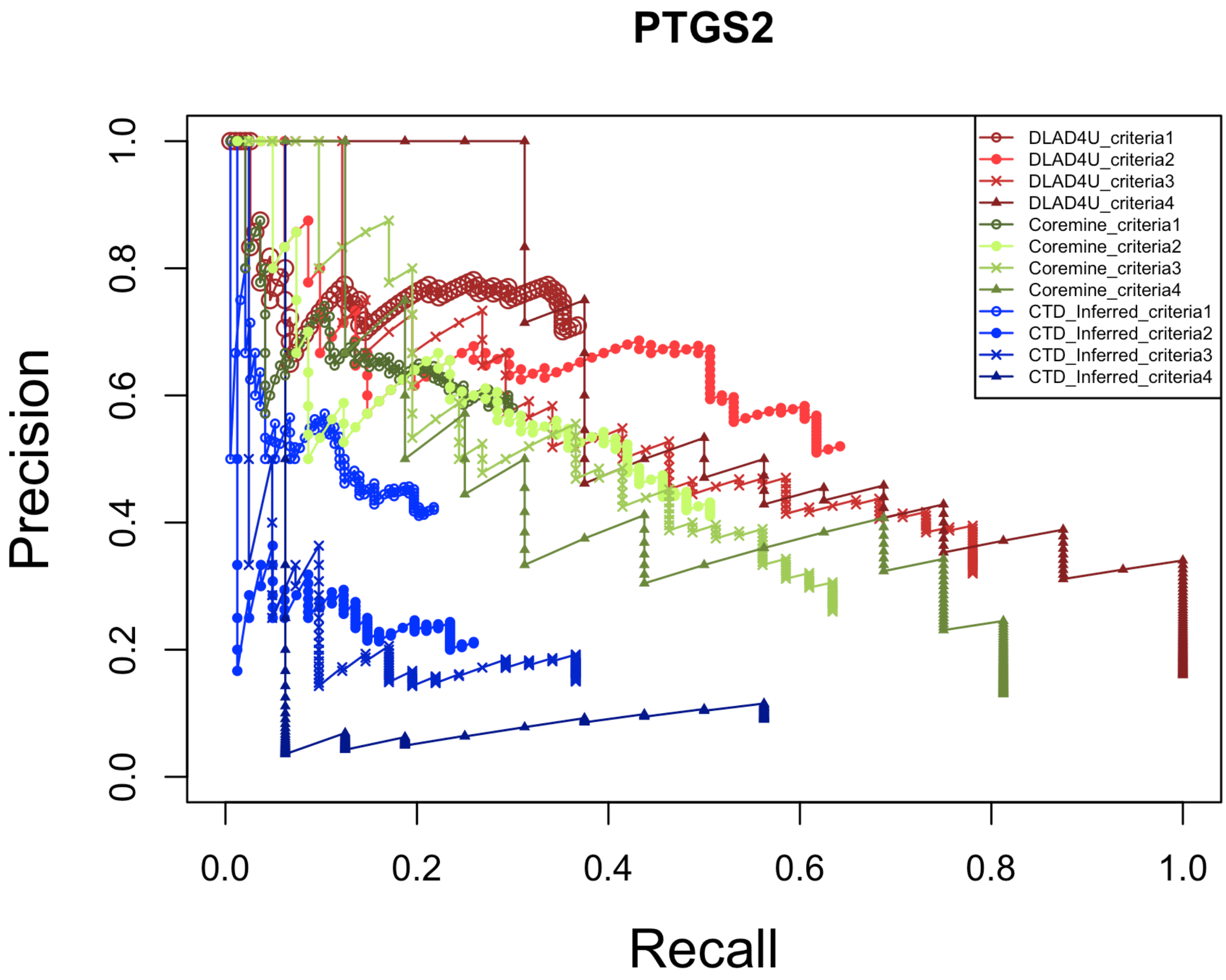

Figure S6. Precision/recall curves for PTGS2 gene. Precision/recall curves for DLAD4U, COREMINE and CTD_inferred are colored in red, green and blue respectively. Different patterns are used to distinguish different criteria.

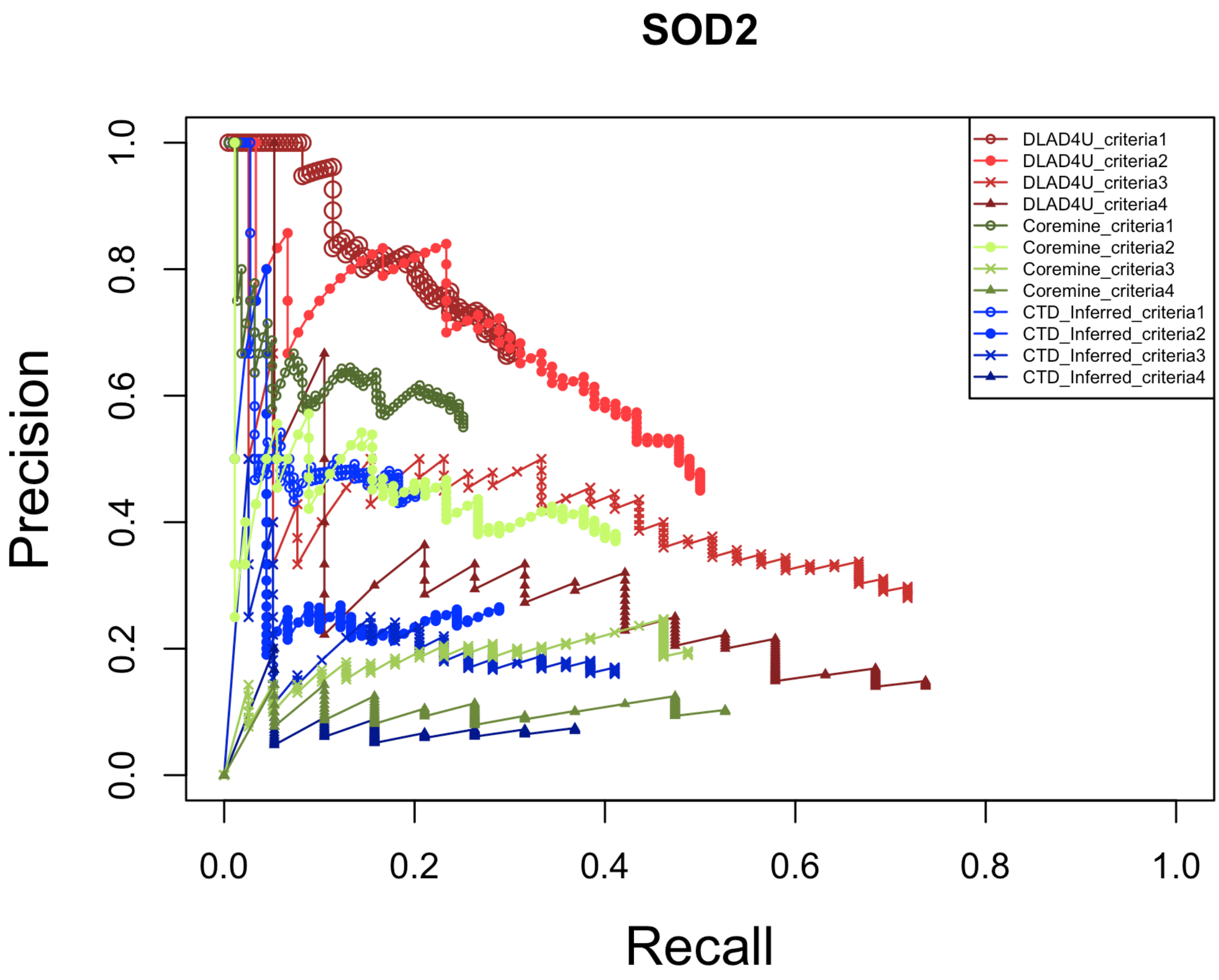

Figure S7. Precision/recall curves for SOD2 gene. Precision/recall curves for DLAD4U, COREMINE and CTD_inferred are colored in red, green and blue respectively. Different patterns are used to distinguish different criteria.

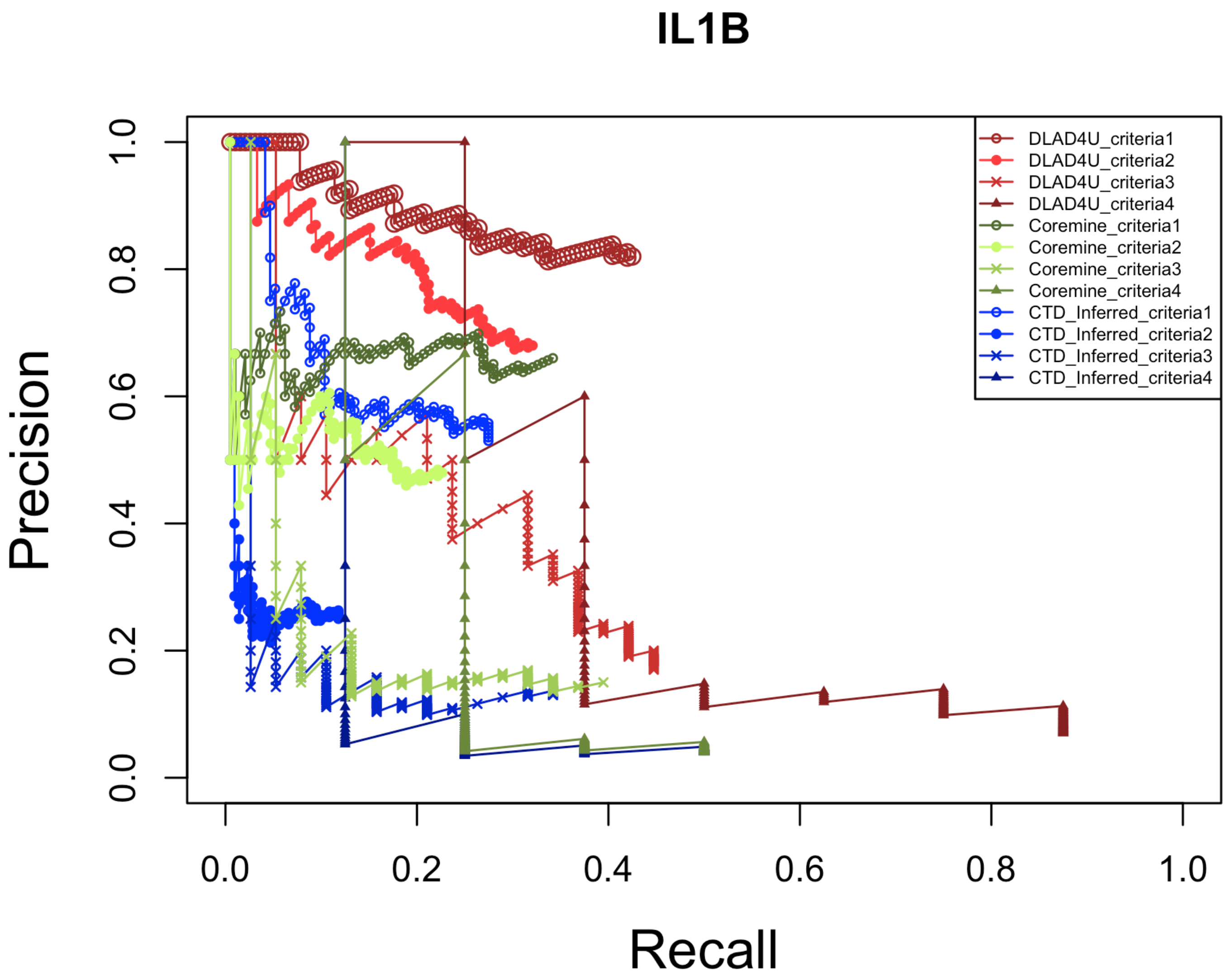

Figure S8. Precision/recall curves for IL1B gene. Precision/recall curves for DLAD4U, COREMINE and CTD_inferred are colored in red, green and blue respectively. Different patterns are used to distinguish different criteria.
